# Environmental Problems: An Analysis of Students’ Perceptions Towards Selective Waste Collection

**DOI:** 10.3389/fpsyg.2021.803211

**Published:** 2022-01-20

**Authors:** Vasile Gherheş, Marcela Alina Fărcaşiu, Iulia Para

**Affiliations:** ^1^Department of Communication and Foreign Languages, Politehnica University of Timisoara, Timis̨oara, Romania; ^2^Department of Marketing and International Economic Relations, West University of Timisoara, Timis̨oara, Romania

**Keywords:** environmental problems, selective waste collection, circular economy, waste management, students, reduce, reuse, recycle

## Abstract

The reduction, reuse, collection and recovery of recyclable materials are sustainable behaviors and people’s awareness of them plays an important role in implementing strategies and policies in this field. The quantitative analysis performed on a group of 816 students of Politehnica University of Timisoara, aimed at finding answers to important environmental concerns and observing the students’ behaviors of reuse and selective collection of the waste resulted from plastic containers, paper, aluminum, batteries, iron packaging waste, electronic equipment, used cooking oil and printer toner. The research has shown that ‘increased amounts of waste’ (63.5%) is among the first three concerns Romania has to deal with, besides ‘air pollution’ (67.9%) and ‘deforestation’ (63.7%). Moreover, the study highlights the existence of the behavior toward the selective waste collection among students (plastic – 60.3%, paper – 57.8%, and glass – 55.3%). although there are some areas (e.g., selectively collecting used cooking oil or printer toner, their level of knowledge regarding the color code for the recycling bins, etc.) that students still need to be familiarized with through different campaigns, trainings, courses, etc. The results can be used in the development of institutional strategies or of strategic documents targeting environmental protection and sustainable development.

## Introduction

Industrialization and an increase in living standards have led to the generation of impressive amounts of waste that, unfortunately, affect the environment through climate change, through their negative impact on fauna and flora and, ultimately, through their impact on our health. By decomposition, the waste from landfills emits methane, a gas over 80 times more powerful than carbon dioxide (Environmental Defense Fund, 2021), and its illegal burning releases high levels of carbon dioxide into the atmosphere; both are greenhouse gases that warm the planet and change the climate. It has been observed that open landfills let out 91% of all the methane emissions from landfills and that about 40% of the world’s waste is burned in this way (Earth Day, 2021). Also, these gases represent unseen dangers for the population in the long run, causing diseases such as asthma, cancer, cardiovascular diseases, genetic disorders in newborns, low birth weight, infectious diseases, etc. Last but not least, due to the ingestion of plastic and garbage, many species of animals, birds and marine mammals are affected, their stomachs being unable to digest the ingested objects.

The Environmental Protection Agency of the United States calculated that, in 2017, the total generation of municipal waste was of 267.8 million tons, a figure that increased by 5.7 million compared to 2015 (Earth Day, 2021), while in 2019, in the European Union, 225 million tons of municipal waste were generated, i.e., 502 kg per person, slightly more than in 2018 (495 kg). Per capita, Denmark (844 kg) was the country that generated the most significant amount of municipal waste in 2019, while Romania ranked last (280 kg) (Eurostat, 2021).

In the EU, environmental efforts have been intensified by implementing initiatives that could lead to a climate-neutral Europe by 2050. Special attention is paid to the circular economy, which aims to reduce waste, and ultimately to reduce its impact on the environment, production and consumption, bringing thus benefits to both the society and the people. Even though circular economy, a cutting-edge research topic both for theoreticians and practitioners (Geissdoerfer et al., 2017), has different definitions, the most used one refers to the activities of reduce, reuse and recycle for economic prosperity and environmental quality (Kirchherr et al., 2017). Circular economy transforms goods and products that are no longer in use into future resources for other people, leading thus to waste minimization (Stahel, 2016), having environmental, economic and social implications both for the industry and for the consumers (Meseguer-Sánchez et al., 2021).

In March 2020, the European Commission put forward a new Circular Economy Action Plan, which focuses on waste prevention and management and aims at boosting economic growth and competitiveness, and at maintaining the Union’s leadership position in this field (Parlamentul European, 2021).

The fact that the basic policy for good waste management should be centered around the 3Rs (Reduce, Reuse, and Recycle) principle is very well-known. Mainly, attention should be paid to reducing the amounts of waste, before generating them, and then trying to reuse them or, if this is not possible, selectively collecting them for recycling. In 2018, in the EU, the recycling of municipal waste increased to 67 million tons, corresponding to 150 kg per capita, three times more than in 1995 (23 million tons) (Eurostat, 2020).

However, things are not going well at all for Romania, which in May 2020 was threatened with the start of an infringement procedure by the European Commission, as it has not made any progress since 2014 in municipal waste management and has not complied with the Court of Justice’s decision of October 18, 2018, to close the 48 illegal landfills, left out of the initial 68, in the important cities in Romania, representing real dangers for the population’s health (European Commission, 2020b).

According to the Country Report on Romania 2020 by the European Commission, Romania is still struggling with waste management, having low recycling of municipal waste (14%) and high landfilling rates (70%) (European Commission, 2020a). Currently, Romania recycles only 15% of the collected waste, with a target of 50%, which is very unlikely to be achieved by 2050 (European Commission, 2019; Florin et al., 2020; Ministerul Mediului, Apelor şi Padurilor, 2021). In line with the revised Waste Framework Directive, that has set more ambitious recycling targets by 2035, the authorities have to ensure that the amount of municipal waste in the landfills is reduced to 10% or less by 2035 (European Commission, 2020a). Measures that could lead to an improvement of the current situation are definitely needed and, for this to happen, people should be made aware of the impact that waste can have on the environment and also of the importance of reducing the amounts of waste that are generated and of the benefits of reusing and recycling them, especially since it was reported that, in the top of the European pollution-related deaths, Romania ranks third with 19% after Bosnia-Herzegovina and Albania (Balkan Insight, 2020).

The transition toward a circular economy depends on the way individuals and organizations adopt values and behaviors that aim to achieve the goal of “zero waste” and turn them into environmentally-conscious consumers, and the importance of sustainability at the local, national and international level. But, in order to attain this, these habits have to be known so that people can understand how their behavior damages the environment, the starting point to obtain education in the circular economy being represented by environmental education initiatives that ensure the development of knowledge, values and attitudes that lead to actions in this respect. In Romania, there are few studies on how people should reuse and recycle and this is a prerequisite for policy development and for a shift toward sustainable behavior. There are also few studies on the young people’s attitude toward environmental problems and on their ecological behavior. Research on pro-environmental behavior, whose determinants can be defined by applying theories such as value-belief-norm, theory of planned behavior, Campbell paradigm, is becoming increasingly important in solving environmental problems and achieving the goals of sustainability.

This research aims at gaining insight into the students’ level of knowledge about the current environmental problems and into the way they adopt sustainable behaviors in a circular economy in order to issue a warning, if necessary, for the need to create educational programs and to inform them about the philosophy of the 3Rs. Students make up an important percentage of the young and educated population and, therefore, they should be the ones setting up the future trends as far as environmental protection is concerned. They could be of help in educating their colleagues and other categories of the population about environmental behaviors and could act as agents of change in both their homes and their future jobs. Due to the fact that they will be the ones who will hold key positions in society, they will be able to contribute to the adoption of sustainable strategies that will lead to the protection of finite resources and of the environment and to putting an end to climate changes.

The research questions were as follows:

• RQ_1_: What is the students’ perception on the environmental problems in Romania, on their degree of importance and on the persons responsible for solving them?
• RQ_2_: What are the reuse behaviors among the study population?
• RQ_3_: What are the behaviors of selectively collecting waste and what is the frequency with which students selectively collect waste resulted from plastic containers, paper, glass, aluminum (beverage cans, cans, etc.), batteries, iron packaging waste, electronical equipment and household appliances, used cooking oil, and printer toner?
• RQ_4_: What is the respondents’ level of awareness regarding the type of waste that has to be collected into the containers for selective collection (based on the color of their lids)?
• RQ_5_: Are there significant differences between the female and the male respondents regarding the studied aspects?

This study aims at providing answers to the above-mentioned questions and at identifying the areas requiring actions to be taken in order to ensure a better reuse and selective collection leading thus to environmental protection. By being aware of certain environmental protection behaviors, but also of the existing problems in this field, stakeholders can develop policies and action plans that could lead to an improvement of the local and regional ecological protection. This research does not aim at answering “why” these behaviors exist; instead, it tries to capture them, as a photograph of the reality we live in, such analyses not having been carried out so far on this category of population and on this topic.

## Literature Review

### The 3Rs: Reduce, Reuse, and Recycle

In order to protect the environment, to reduce pollution and to save natural resources, it is necessary to reduce waste, to reuse or to recycle it. These actions are known as the 3Rs, Reduce, Reuse, and Recycle, which, from the mere marketing slogan, have become a way of life for many people.

Reduce refers to the awareness of the fact that we do not need all the products we use, from clothes to food. Consuming less means consuming better and the emphasis should be on a qualitative approach, not on a quantitative one. We should also ask ourselves whether these products with a short life cycle, which will become waste very quickly, really contribute to our well-being and happiness or are just a whim. According to the Institute for European Environmental Policy, the way we consume today is not sustainable; by 2050, every European will have to reduce by 80% the natural resources they currently use for nutrition, accommodation, mobility and pleasure. This thing can only be achieved through a combination of efficiency and sufficiency (Institute for European Environmental Policy, 2019).

Reuse, means finding another use for a specific thing that was to be thrown away and thus increasing its value, making sure that it does not end up in the landfill or recycling center. Before throwing a product in the trash, we should ask ourselves if it can be reused or repaired, saving in this way energy, time, money, resources and eventually ensuring the environmental protection. In other words, each of us should make an effort and consume as few disposable materials as possible. Reports have been written on great business opportunities for companies looking to reuse plastic (Ellen MacArthur Foundation, 2017; Resource Recycling, 2019). Ellen MacArthur Foundation provides a general theoretical model of circular economy based on maintenance, reuse, recovery and recycling of the high volumes of production and waste (Ellen MacArthur Foundation, 2017), while other researchers (Lewandowski, 2016; Silveti and Andersson, 2019), also emphasize the importance of reuse and of the fact that products are created to have a longer life cycle and that companies focus mainly on the reuse of their products, extending thus their life cycle through maintenance, repairs and sustainability.

Recycling helps to conserve resources and reduce the production costs of many products and comprises the process of selective collection (Bacău et al., 2021). The amount of recycled waste has increased a lot, almost tripling from 37 million tons (87 kg per person) in 1995–107 million tons (239 kg per person) in 2019. The amount of municipal waste incinerated in the EU has doubled from 1995 from 30 million tons (70 kg per person) to 60 million tons (134 kg per person) in 2019 (Eurostat, 2021).

Throughout the time, studies have been undertaken in various fields, e.g., medicine (Kirkby, 1993), textile industry (Weber et al., 2017), electronic waste or e-waste (Cairns, 2005; Terazono et al., 2006; Dempsey and Palilonis, 2012), construction waste (Tam and Tam, 2006; Kabirifar et al., 2020) or household waste (Barr et al., 2001), in order to observe the implementation of the 3Rs in waste management.

### Selective Collection of Waste in Romania

The selective collection as part of the separate collection is also an essential part of the process of waste recycling to be introduced into the economic cycle (Ciuta et al., 2015). The need for selective collection in order to recycle waste and capitalize on it is increasingly important, due to the fact that Europe is a continent that is poor in raw materials and mineral resources, the European industry relying heavily on imports. For the European countries, there is a European Parliament requirement to carry out concrete measures in order to raise the competitiveness of the secondary raw materials, thus prohibiting the deposit of waste on landfills and ultimately reaching the “zero waste” status (Marcu, 2010; Burlakovs et al., 2018).

The selective collection has at least three objectives: (1) protection of the population’s health; (2) protection of the environment; (3) protection and conservation of natural resources. The waste management options aim at preventing the occurrence of waste in waste-generating activities through clean technologies, at reducing the amounts by applying the best technologies, at recovering through reuse, material recycling and energy recovery. As part of the European Union, Romania has signed the Environmental Agreement, which is found in Chapter 22 of the Treaty of Accession to the European Union. Furthermore, based on the European and national legislation provisions in the field, Romania has developed and approved the National Waste Management Plan, which aims at creating the necessary framework for developing and implementing an integrated waste management system, efficient from an ecological and economic point of view and which details the structure of the waste [mixed household waste from the population; assimilable mixed waste, from trade, industry, institutions; municipal and assimilable waste collected separately (with the exception of construction and demolition waste); bulky waste; green waste; market waste; road waste; generated and uncollected waste]. The selective collection is carried out mainly for the household mixed waste collected from the population, for the assimilable mixed waste collected from trade, industry, institutions, for the municipal and assimilable waste collected separately (with the exception of construction and demolition waste). The selective collection is carried out on several fractions or in a dual system, by wet and dry garbage. The selective collection on several fractions is mainly implemented in the developed countries of the EU, but it has proven to be more expensive; in Romania, waste is usually collected in a dual system, by wet garbage (organic, biodegradable materials, infected paper and cardboard, textiles, small inorganic materials) and dry garbage (paper and cardboard, plastic, glass, metal, and wood).

Nowadays, waste management treats the population differently from public institutions from the waste collection point of view (Iojă et al., 2012; Ciuta et al., 2015; Izvercian and Ivascu, 2015); the legislation is also adapted, but the general purpose remains the same, i.e., reducing the amounts of waste, reusing or recycling them.

The fundamental legislation on selective waste collection starts from Law 211/2011, which establishes “the necessary measures for protecting the environment and the population’s health by preventing or reducing the adverse effects caused by waste generation and management” (Ministerul Mediului, Apelor şi Padurilor, 2011). The law establishes the following hierarchy in waste management: prevention, preparation for reuse, recycling, recovery and disposal. Also, under articles 16 and 17, the same law stipulates the obligation of the central public administration authorities governing the environmental protection and of the local public administration to ensure the separate collection for at least the following types of waste: paper, metal, plastic and glass. According to the regulation in practice, Law 211/2011 for households and blocks of flats and Law 132/2010, amended and supplemented by Law 194/2019, on the selective collection of waste in public institutions, all over the territory of Romania waste is collected in different colored bins for institutions and two differently colored bags for households (black bag and yellow bag). Under GEO 74/2018, starting with July 01, 2019, the selective collection of waste is mandatory on four fractions: plastic/metal, paper/cardboard, glass, residual/mixed/household waste. For blocks of flats and individual households (houses), containers/bins or yellow bags will be used as follows: for houses - in specifically colored plastic bags provided free of charge by the sanitation operator -, and for blocks of flats – in yellow bins (plastic/metal), blue (paper/cardboard), green (glass), and black (residual waste) (Ministerul Mediului, Apelor si Padurilor, 2018). However, it is worth mentioning the fact that although there is a clear legislation in this regard for public institutions, for households and blocks of flats the situation remains the same, in two different colored containers, as there are no methodological norms from the central authorities to be put into practice. A particular chapter in the selective collection of waste is treated by Law 132/2010, amended and supplemented by Law 194/2019, on the selective collection of waste in public institutions (Portal Legislativ, 2010, 2019). It established the mandatory legal framework for public institutions regarding the selective collection of waste. Thus, enforcing this law will ensure the degree of selective collection and the increase of the degree of awareness, information, education of the employees and the citizens. Public institutions may carry out the selective collection directly or may delegate this responsibility to third party operators. Each public institution shall appoint a person in charge by the decision of the head of the institution. The legislation stipulates that a plan of measures should be drawn up regarding the selective collection of its waste in each public institution. Also, the employees will be trained through a pre-established program, and an informative program will be prepared for the visitors. Moreover, the waste will be recorded and weighed upon delivery by writing it in a register.

For the population, to stimulate selective collection, local public administrations should apply the principle “pay for how much you throw away,” this becoming mandatory only in 2021, under Law 181/2020, in accordance with which all biodegradable waste must be collected separately from other waste, in brown bins or converted into natural fertilizer in your household, with the help of special containers for individual composting (Portal Legislativ, 2020).

Reporting data at the national level on the amounts of waste generated and/or collected differs significantly from one source to another (see data reported by ADID versus the County Waste Management Plan). Moreover, there is no official data on the amounts of waste before 1997 (according to Eurostat, in 1997, Romania reported 325 kg of municipal waste/year/inhabitant and in 2018, 272 kg/year/inhabitant, i.e., decreasing) (Eurostat, 2020).

### Selective Collection of Waste in the Timis County

With respect to the Timis County, the county where the university whose students that were surveyed is located, from the amount of municipal and assimilable waste from trade, industry and institutions that was generated and collected at the county level and at all the territorial-administrative units, respectively, in accordance with the County Report on the state of the environment for the year 2016–2019 of the Timis county (Ministerul Mediului, Agenţia Naţională pentru Protecţia Mediului, 2020), the following results were obtained: a significant increase in the amounts for the period 2012–2018, from 116,711.5 to 180,547 tons. Thus, in the Timis county, the amount of selectively collected municipal waste (tons) in 2012 was 11,686 tons, and in 2018, 28,023 tons. The results confirm an increase of almost 54% for 6 years, and what deserves special attention is the fact that approximately 205% is the increase of the amounts collected selectively at the level of all the territorial-administrative units within the county since, in the period prior to 2012, the selective collection in the Timis county was carried out exclusively in Timisoara. Starting with 2012–2013, the selective collection of waste was progressively implemented on the entire territory of the Timis County, which led to this increase of 205%.

To deepen the research, information on selective collection from the Timis Waste Intercommunity Development Association (ADID Timis), which is the coordinating body for municipal and similar waste collection in the Timis County, Romania, was requested. The information received following the request has been approved to be processed for academic and research purposes. The data obtained from ADID Timis refer strictly to the period 2019–2020, the information not being made public until now. Nevertheless, after having processed the transmitted data, the following aspects regarding the period 2019–2020 can be drawn. First, at the level of the Timis County, the percentage of selectively collected waste from the total collected waste is 15.6%, of which in urban area is 18% and in rural area 11%, the conclusion being that in the urban area, the selective collection is much more advanced with regard to the implementation and the effective collection, compared to the rural area. Second, the percentage of 15.6% comes from the ratio of the amount of recyclable waste plus glass divided by residual waste plus recyclable waste plus glass. Therefore, the percentage means the amount of recycled waste from the total amount collected. The same formula applies to data on urban and rural areas.

Compared to the targets set by the National Plan and the County Waste Management Plan, targets assumed and agreed with the EU, it can be observed that both Romania as a country and the Timis County are at a considerable distance, reaching so far a higher percentage of 18%, compared to the set target of 50%.

As far as the collection points are concerned, there are approximately 538 selective collection points for glass in Timisoara; general collection points for the population do not exist, but instead, there are collection points for waste generators (population, blocks of flats, private households, state institutions, and companies). There are only street waste collection bins at the local level, but they are not especially designed for the selective collection, as they are not in the recreational areas or in the university campus. The concept of selective collection established by the Local Waste Management Plan was to equip the waste generator with a garbage can/yellow bag for houses, blocks of flats and commercial companies for the selective collection. Inside the institutions, the selective collection system is organized according to national and European regulations, but the collection performed by the sanitation operator is performed strictly from the yellow bin in which both plastic and cardboard, paper and aluminum are mixed.

In Timisoara, since 2011, there is a functional municipal waste sorting station, the selectively collected waste is manually sorted on conveyor belts in at least four assortments: cardboard + paper, PET, aluminum cans, PPD. The rest of the waste collected in the black bin is also sorted in this sorting station, in two fractions, i.e., in the dry fraction that can be recovered energetically at the cement factories and in the wet fraction that is deposited at the landfill called the Integrated Management Center of Municipal Waste in Timis County, located in Ghizela.

The sorting of separately collected DMS fractions is applied internationally. The process is performed before treatment and storage. The procedure proved to be a good method of meeting the waste targets in the packaging, given that sorting the DSM collected in the mixture proved difficult and disappointing. In the municipal waste sorting technology, the main aim is to increase the pre-treatment of materials previously separated from municipal solid waste by screening processes to increase the efficiency of manual sorting. The process can reach up to 220 kg/h for paper and cardboard and 145 kg/h for light fractions such as PET bottles or other plastic products. Ferrous and non-ferrous metals are generally removed by magnetic separators. The municipal waste sorting station in Timisoara functions as a recycling center that collects and capitalizes approx. 60–70% of the municipal waste of Timisoara municipality. In light of these statistics, this study aims at shedding light on the way students view environmental problems and on their behaviors on reuse and selective collection. In a constantly developing world, students represent the young and informed segment of the population that will create a more sustainable world in the future.

## Materials and Methods

In order to conduct the research, a quantitative analysis was carried out, and in order to collect the data, a questionnaire was applied. The target population was represented by the students of Politehnica University of Timisoara and the data were collected during March–April 2021. The students of this university were chosen for this study due to the fact that the authors were able to communicate with them during the COVID-19 pandemic, an extension of the study to other universities in the country being almost impossible during this emergency time. The study was carried out on a sample of 816 subjects from all the study years. As the university’s student body counts around 13,000 students, the calculated margin of error was of ±3.3%. Starting from the assumption that a student’s gender may impact their behaviors and attitudes, based on existing studies that have already emphasized that (Vicente-Molina et al., 2018; Ramstetter and Habersack, 2020; Wut et al., 2020), the sample was built taking into account the fact that the respondents’ gender distribution should be a relatively equal one as the aim of the study was to identify certain particularities of the answers related to this variable; being one of the research questions, it was used for the secondary analysis of this research. Thus, a sample of 409 females and 407 males was built. Their average age, according to the recorded results, was 20.37 years old.

A questionnaire was used to collect the data, its content being validated through the following steps: assessment by experts (sociologists) followed by its qualitative and quantitative pretesting. The Cronbach’s Alpha test was used to test the internal consistency of the Likert scales used in the questionnaire. Closed-ended and open questions were used in the questionnaire and, at the end, factual data regarding gender, age, year of study, rural or urban background were also requested.

In order to answer the first two research questions that aimed to capture the students’ perception of environmental problems in Romania, their importance and the people responsible for solving them, five questions were included in the questionnaire (four closed questions and one open question). In order to obtain answers to the third research question (reuse behaviors among the study population), six scale questions were used (5-point Likert scale, Cronbach’s Alpha’s value = 0.758), and nine scale questions (5-point Likert scale, Cronbach’s Alpha’s value = 0.859) were used to capture the selective collection behavior. The Alpha values obtained for the Likert scales are considered good, being higher than the minimum acceptable level of 0.7 for internal consistency (Secolsky and Denison, 2017).

To find out the respondents’ level of awareness regarding the type of waste that has to be collected into the containers for selective collection, five questions with the same answer variants were used, the respondents being asked to choose the correct variant. The data were analyzed using SPSS Statistics, a software package frequently used for statistical analysis. The data analysis was performed based on the frequencies obtained and, in order to provide answers to the last research question (i.e., the gender variable, on which the secondary analysis was based), a chi-squared test was conducted for all questions.

The questionnaire was anonymous and was applied online on the Isondaje.ro platform (an online survey service). Due to the conditions imposed by the COVID-19 pandemic limiting face-to-face interactions, the questionnaire was applied online as this was considered to be a safer and a more efficient method, the response rate being of approximately 50% and the average duration of completion of about 15 min. The completion of the questionnaire was voluntary and the students could opt out of filling it in at any time; also, no rewards were used for this activity. For the sake of anonymity and confidentiality, the students’ e-mail addresses or other personal data were not collected.

## Results

### Students’ Perception of Environmental Problems in Romania, of Their Degree of Importance and the People Responsible for Solving Them

One of the questions addressed to the respondents with the purpose of finding out their opinion on this matter was “How important is environmental protection to you*?*”. Although the answer variants that were provided covered the whole range of answers from “very important” to “not quite important,” as it can be seen in the table below (Table 1), most of them centered around two answer variants, i.e., “quite important” (52.5%) and “very important” (45.5%). Taking into account these results, it can be posited that the respondents consider environmental problems as being very important; the fact that the “not quite important” and “not at all important” answer variants were selected by very few of them (2.1% and none of the respondents, respectively) also emphasizes this result.

**TABLE 1:** Cross Tabulation and Chi-Square Test on environmental protection based on gender.

			Gender					
			Male	Female	Total	Chi-square	DF	Sig.
How important is environmental protection to you?	(1) Not quite important	*F*	12	5	17	17.018	2	0.000
		*P*	2.9%	1.2%	2.1%			
	(2) Quite important	*F*	238	190	428			
		*P*	58.5%	46.5%	52.5%			
	(3) Very important	*F*	157	214	371			
		*P*	38.6%	52.3%	45.5%			
Total		*F*	407	409	816			
		*P*	100%	100%	100%			

*A value of χ^2^ = 17.018 and a value of p = 0.00 (p < 0.05) were recorded, showing that the female respondents are more interested in the environmental problems than the male respondents.*

In order to point out other aspects regarding the importance of the environmental protection, a secondary analysis was carried out in order to verify whether there are significant differences among the study population related to the respondents’ gender. Following the application of the chi-squared test, the results show that there are significant differences between females and males concerning the way environmental problems are viewed (Table 1).

**FIGURE 1:**
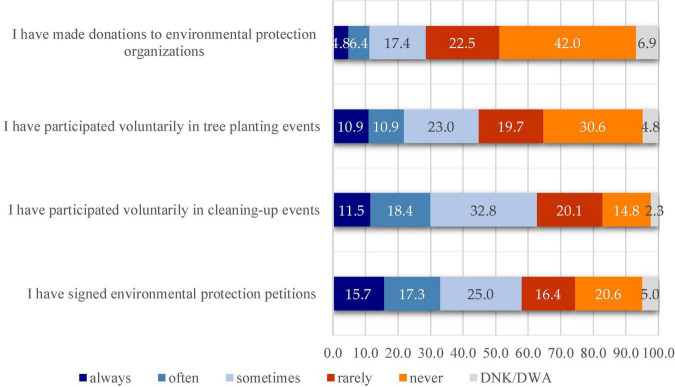
Students’ involvement in environmental protection events.

Closely related to the above question was also the question seeking to identify the extent to which the study subjects were involved in environmental protection actions. As it can be seen from the graph below (Figure 1), very often, the surveyees signed environmental protection petitions (33% by cumulating percentages from the “always” and “often” answer variants) and participated voluntarily in cleaning-up events (29.9%).

The existence of significant differences between the male and female respondents was also tested in this case. Following the application of the chi-squared test, such significant differences were recorded only for two of the four above-mentioned statements as it can be seen in Tables 2, 3. Based on these results, the following observations can be made:

• There are significant differences between females and males in the way they “have participated voluntarily in cleaning-up events” (Table 2). A value of χ^2^ = 35.446 and a value of *p* = 0.00 (*p* < 0.05) were recorded. The results show that this type of behavior is more specific to females than to males.
• There are significant differences between females and males in the way they “have signed environmental protection petitions” (Table 3). A value of χ^2^ = 40.845 and a value of *p* = 0.00 (*p* < 0.05) were recorded. The results show that this type of behavior is more specific to females than to males.

**TABLE 2:** Cross Tabulation and Chi-Square Test on the voluntary participation in cleaning-up events based on gender.

			Gender					
			Male	Female	Total	Chi-square	DF	Sig.
I have participated voluntarily in cleaning-up events	(1) Never	*F*	81	40	121	35.446	5	0.000
		*P*	19.9%	9.8%	14.8%			
	(2) Rarely	*F*	91	73	164			
		*P*	22.4%	17.8%	20.1%			
	(3) Sometimes	*F*	130	138	268			
		*P*	31.9%	33.7%	32.8%			
		*F*	65	85	150			
	(4) Often	*P*	16.0%	20.8%	18.4%			
		*F*	28	66	94			
	(5) Always	*P*	6.9%	16.1%	11.5%			
Total		*F*	407	409	816			
		*P*	100%	100%	100%			

**TABLE 3:** Cross Tabulation and Chi-Square Test on signing environmental protection petitions based on gender.

			Gender					
			Male	Female	Total	Chi-square	DF	Sig.
I have signed environmental protection petitions	(1) Never	*F*	108	60	168	40.845	5	0.000
		*P*	26.5%	14.7%	20.6%			
	(2) Rarely	*F*	83	51	134			
		*P*	20.4%	12.5%	16.4%			
	(3) Sometimes	*F*	94	110	204			
		*P*	23.1%	26.9%	25.0%			
		*F*	58	83	141			
	(4) Often	*P*	14.3%	20.3%	17.3%			
		*F*	43	85	128			
	(5) Always	*P*	10.6%	20.8%	15.7%			
Total		*F*	407	409	816			
		*P*	100%	100%	100%			

Another aspect this study has focused on was identifying the respondents’ opinion regarding Romania’s main environmental problems. To this purpose, two questions were added to the questionnaire: an open one, the answers to which were coded after the end of the survey, and a closed-ended one, comprising a predefined list of problems from which the subjects had to choose the top three environmental problems by order of preference.

**FIGURE 2:**
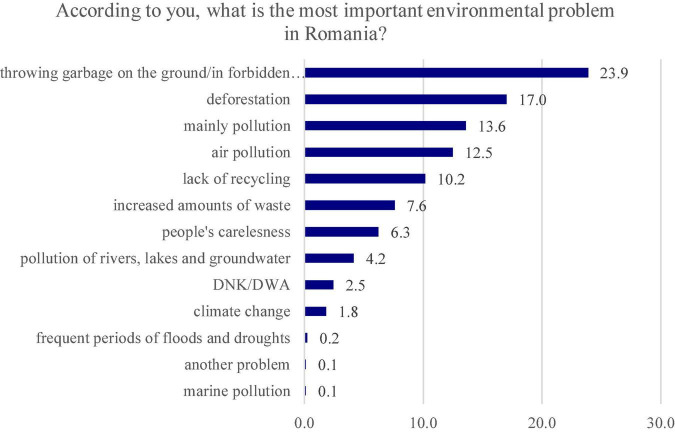
The most important environmental problems in Romania according to the respondents.

By analyzing the results obtained for the open question (Figure 2), depending on the percentages received, “throwing garbage on the ground/in forbidden places” is considered the main environmental problem in Romania (23.9%), being followed by “deforestation” with 17% and “mainly pollution” with 13.6%.

For some answer variants, this question has also presented some small differences between the female and male respondents (resulted from the association tables), but they are not statistically significant. As trends, it can be seen that the biggest differences are registered in the case of the “deforestation” answer variant where, compared to the average obtained from the whole group of respondents, which was 17%, there were variations of up to 21.9% for males and of up to 12.2% for females. Another answer variant for which the statistical differences are not significant but for which there were deviations from the average value (13.8%) is the “mainly pollution” answer variant, with recorded values of 20.3% for the female population and 6.8% for the male population. For the other answers, the differences that were recorded were much smaller.

**FIGURE 3:**
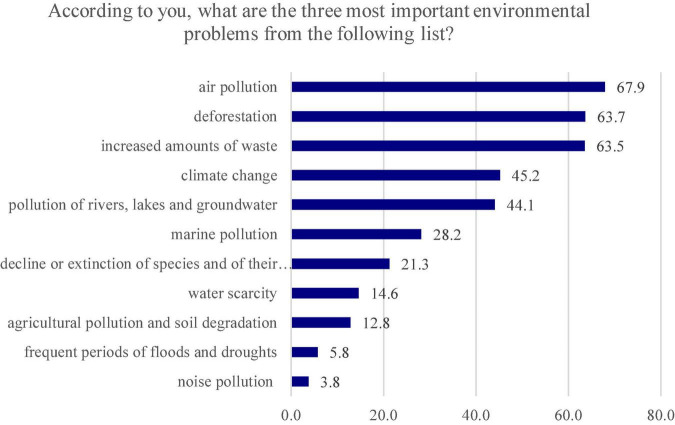
The three most important environmental problems according to the respondents.

The answers obtained for the closed-ended question provided a different hierarchy, the first three places being taken by “air pollution,” which cumulated 67.9% of the answers, “deforestation” (63.7%) and “increased amounts of waste” (63.5%) (Figure 3). By comparing the results of the two questions, it can be posited that the problem of “deforestation” ranks 2nd in both hierarchies, the other problems being slightly different.

**FIGURE 4:**
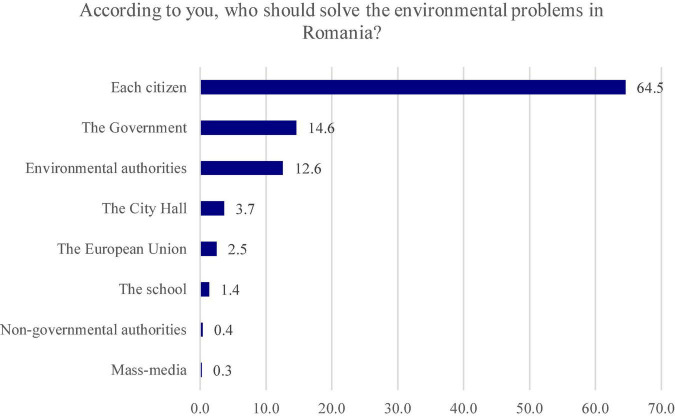
The main actors in solving environmental problems according to the respondents.

According to the respondents, those who should solve the environmental problems are the citizens, this answer variant totaling 64.5% of the responses. At a great distance, there are also the surveyees who consider that “The Government” (14.6%) and the “Environmental authorities” (12.6%) are responsible for dealing with the environmental problems (Figure 4).

### Students’ Reuse Behaviors

**FIGURE 5:**
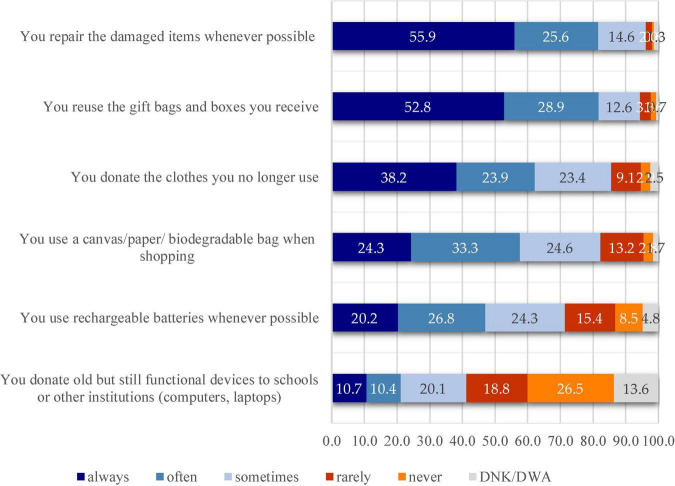
Percentages recorded for the reuse behavior.

The process of waste generation cannot be stopped, but each person can have a positive impact in this matter if they decide to bring around an individual change. By implementing the principle of the 3Rs (Reduction – Reuse – Recycle), people can show they care for the environmental problems that are being felt more and more every day, but also for the conservation of natural resources. Being an integral part of the ecological behavior, reuse is one of the very important steps in the list of actions that each person can take so as to generate as little waste as possible to ultimately reach the landfill. Another aspect the study has addressed was capturing the reuse behavior since nowadays this activity is becoming a trend. Many times, people tend to use things only once and then throw them away. By reusing the materials considered as waste, people can show they care about the climate change and can stop overusing the natural resources by reducing the amount of waste, by reusing it or by donating it to others. As it has been explained in the introductory part of the paper, in order to capture this behavior, six statements referring to the behavior of reuse were added to the questionnaire. The Figure 5 shows that the statement that recorded the highest percentages for the “always” answer variant was “You repair the damaged items if possible,” answered by more than half of the respondents (55.9%). In the order of the recorded percentages, the “often” answer variant follows with 25.6%.

Another statement that exceeded 50% for the “always” answer option was “You reuse the gift bags and boxes you receive” (52.8%), being followed by the category of the respondents who chose the “often” answer option (28.9%). If we were to consider that we can talk about the existence of a reuse behavior among the respondents for the situation where they choose the “always” and “often” answer variants for the statements under investigation, then, the two situations described above fit perfectly to this pattern, the cumulative scores exceeding 80%. “You donate the clothes you no longer use” ranked 3rd in this hierarchy, scoring only 38.2% for the “always” answer option. This is also a case in which we can talk about the existence of a behavior among the surveyees because, taking into account the same principle of cumulation of the scores obtained for the two answer variants, it is clear that a score of 62.1% is obtained. “You donate old but still functional devices to schools or other institutions (computers and laptops)” ranked last in this hierarchy of reuse behavior, scoring only 21.1% by cumulating the two answer variants.

### Students’ Selective Collection Behavior

Another objective of this study was to observe the students’ behavior of selective waste collection. The fact that energy is saved, that natural resources are conserved, that pollution decreases by reducing carbon dioxide emissions and, thus, the greenhouse effect, that the amounts of garbage are reduced, that cheaper packaging and products are obtained, ultimately leading to an increase in the quality of life, can be mentioned among the advantages of selective collection. In this research, the behavior of selective collection is discussed from a general point of view, without emphasizing its occurrence in clearly defined settings, such as at home or in educational institutions. Although, in Romania, under the enforceable legislation, the individuals, the public institutions, the companies, the associations, the foundations, i.e., all the people residing in Romania, have the duty and legal obligation to selectively collect waste, the statistics rank us among the last European Union countries in this respect.

To better comprehend the behaviors of selective collection and to better view the results, the percentages obtained for the “always” and “often” answer variants (variants that can lead to the idea of existence of such a behavior), were cumulated.

**FIGURE 6:**
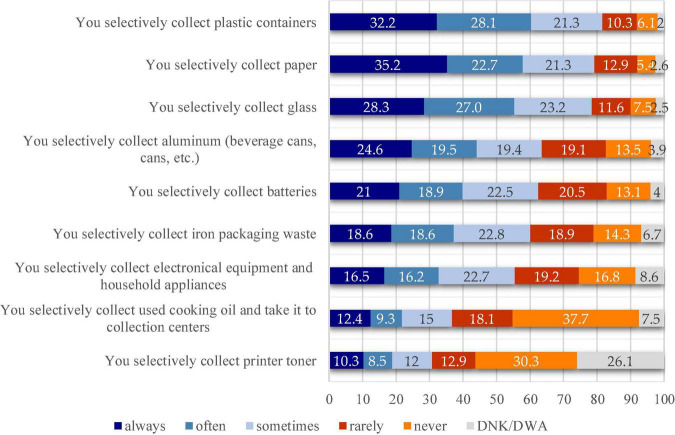
Percentages recorded for the behavior toward selective waste collection.

As it can be seen in the Figure 6, in the ranking of selective collection, the first place is taken by the collection of plastic containers that obtained a cumulative score of 60.3%, followed by the collection of paper, with a total of 57.8%. Abiding by the same principle of cumulating the scores obtained for the two answer variants, it can be posited that the selective collection of glass ranks 3rd with 55.3%.

The answer variants presented above are those exceeding 50%. At the other extreme, the collection behaviors that scored high values for the “never” and “rarely” answer variants can be found. “You selectively collect used cooking oil and take it to collection centers” scored the highest percentages, 53.8% of the respondents saying they do so “rarely” or “never” (score obtained by cumulating the two answer variants). This behavior is followed by “You selectively collect printer toner”; in case the above principle of calculation is used, a score of 43.2% is obtained. This last statement also recorded the highest values for the “DNK/DWA” (Don’t know the answer/Don’t want to answer) answer variant with 26.1%.

Following the application of the chi-squared test, the results show that there are significant differences between males and females in the way they collect iron packaging waste (Table 4). A value of χ^2^ = 13.939 and a value of *p* = 0.01 (*p* < 0.05) were recorded. The results show that this type of behavior is more specific to males than to females.

**TABLE 4:** Cross Tabulation and Chi-Square Test on selectively collecting iron packaging waste based on gender.

			Gender					
			Male	Female	Total	Chi-square	DF	Sig.
You selectively collect iron packaging waste	Never	*F*	44	73	117	13.939	5	0.016
		*P*	10.8%	17.8%	14.3%			
	Rarely	*F*	72	82	154			
		*P*	17.7%	20.0%	18.9%			
	Sometimes	*F*	92	94	186			
		*P*	22.6%	23.0%	22.8%			
		*F*	88	64	152			
	Often	*P*	21.6%	15.6%	18.6%			
		*F*	85	67	152			
	Always	*P*	20.9%	16.4%	18.6%			
Total		*F*	407	44	73			
		*P*	100%	100%	100%			

For the other answer variants, no significant differences related to the respondents’ gender were recorded.

### Students’ Level of Awareness of the Type of Waste Collected Into the Containers

The study also tried to investigate the respondents’ level of knowledge regarding the color codes of the containers for the selective waste collection with two questions. To the first one, “Do you know the color code of recycling bins?”, almost three quarters of the respondents (74.6%) selected “yes.” This was followed by “no” with 21.9% and “DNK/DWA” with 3.6%. Then, the second question, “What are the types of waste selected according to the color code of recycling bins?”, was introduced in the survey in order to capture the respondents’ level of awareness regarding the type of waste that has to be collected into the containers for selective collection (based on the color of their lids). For every color code, the same five answer variants were given, and the respondents had to choose the correct one based on their knowledge. In accordance with the enforceable legislation, the color codes that are used are as follows:

• The yellow bin is used for the selective collection of plastic and metal (foils, HDPE, PET, PVC, and other plastics, but also ferrous and non-ferrous metals).
• The blue bin is used for the selective collection of cardboard and paper (printed paper, mixed paper).
• The green bin is used for the selective collection of glass (colored glass and white glass).
• The gray/brown bin is used for the collection of the mixed fraction (bio-waste and household waste),
• The black bin is used for the collection of biodegradable waste from one’s own household (dried leaves, grass, flowers, vegetable, and fruit leftovers).

**FIGURE 7:**
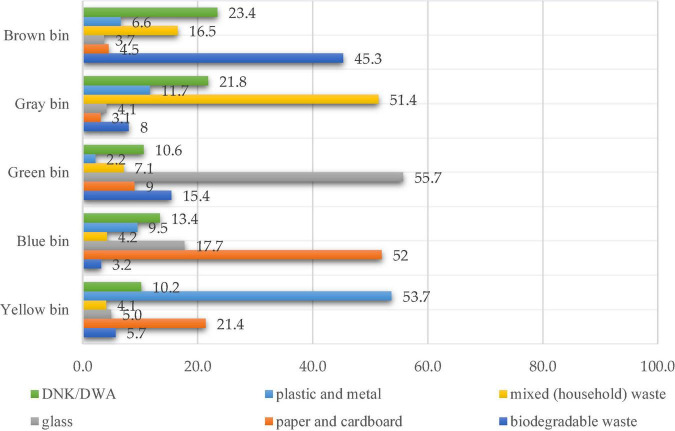
Percentages recorded for the respondents’ level of knowledge regarding the color code for the recycling bins in Romania.

As it can be seen in the Figure 7, the correct answers provided by the respondents to the second question (mentioned above) are different from the ones in the first question, where almost two thirds of the respondents stated that they were familiarized with this color code.

The correct answer variants ranged from 45.3% for the brown bins to 55.7% for glass. At least 10% of the respondents stated, for all the situations under investigation, that they did not know the answer to them. Therefore, it can be concluded that awareness campaigns are definitely necessary to inform the population about the color code used for recycling bins. There were no significant variations in the answers based on the respondents’ gender.

## Discussion

Waste, in addition to being a problem for the environment and generating economic losses, if properly sorted, handed over and recycled, can be used as a resource to create new products. People should be made aware of the impact of waste on the environment, of reusing and recycling it. Although selective collection systems have been introduced directly at the generating source of the waste, they are still not enough due to several reasons, e.g., vandalized collection containers; operators do not follow the collection calendar and mix wastes; large amounts of recyclable waste are stored (Frone and Frone, 2020).

The interest among researchers regarding this topic seems to be high. International studies in universities, based on questionnaires administered to students, have tried to capture the level of the students’ awareness of waste minimization, selective collection, reuse and recycling problems with the purpose of further creating awareness programs, campaigns and strategies or even introducing new study disciplines on waste management and the 3 Rs in the curriculum (Desa et al., 2011; Barloa et al., 2016; Saladié and Santos-Lacueva, 2016). The studies have suggested the fact that the students were aware of the above-mentioned problems, but that more awareness campaigns were necessary to teach them about how to put this knowledge into practice.

In Romania, the studies carried out on this topic have been based mainly on the data provided by the decision-makers and, thus, there are very few of them that are based on surveys. Therefore, the research undergone by Pop et al. (2015), can help us understand the population’s perception and behavior regarding the environmental problems. By way of comparison with this study, the above-mentioned study is based on a smaller number of respondents from other social and educational backgrounds. At the same time, the issue of selective waste collection has also been studied in other counties and on other categories of respondents (Gherheş and Obrad, 2017).

The students’ perception regarding the environmental problems was also surveyed by Târţiu (2011) on 257 respondents, students of ASE University of Bucharest (BAES), who expressed their concern about the environmental problems in Romania and requested additional information to learn about this topic.

In general, the studies are carried out in the Bucharest-Ilfov area, but not only (Târţiu, 2011; Iojă et al., 2012; Sima et al., 2019; Ianoş et al., 2021), while the western part of the country has not been researched regarding the educated young people’s perception on environmental problems.

This category of respondents, i.e., the students of Politehnica University of Timisoara, was included in several other studies, which mainly focused on topics regarding students’ human values (Dragomir et al., 2020) or their attitudes on electricity, water, plastic or paper consumption (Gherheş and Fărcaşiu, 2021; Gherheş et al., 2021) but did not discuss the above-mentioned topic.

As the results show, the subjects of this study consider to a large extent that the environmental problems are important and very important for them. If these results are compared with those obtained by the Eurobarometer in December 2019, the values recorded are close to the average obtained in that year in the EU countries, 94% of the citizens saying that the environmental protection is important for them. This study’s results exceeded this average, the recorded value being 98%, higher than the value obtained for Romania, which was 87% in 2019. The difference could be found in the respondents’ background as the study carried out by the European Commission targeted the entire population. Waste is considered to be one of the biggest environmental problems we face in Romania, a fact confirmed by the answers given to the open question in the questionnaire. To “Throwing garbage on the ground/in forbidden places,” an answer variant which cumulated the highest percentages, “lack of recycling,” and “increased amounts of waste” could also be added as they refer to the same problem, that of waste. “Pollution of rivers, lakes and groundwater” as well as the answer variants that refer to “mainly pollution” or “climate change,” a partial consequence of waste, could also be included here. Even if “increased amounts of waste” was included in a list with other environmental problems, the respondents ranked it on 3rd place, not very far from “air pollution” and “deforestation.” As above, the percentages recorded for the variants referring to waste could be added to “increased amounts of waste.”

It is interesting to note that the secondary analysis that was carried out revealed the fact that gender also seems to play an important part in the respondents’ attitudes toward the importance of the environmental problems in that the female respondents are more aware of the need to protect our environment and also in that they voice and show their concerns more by signing petitions and doing volunteering work as far as environmental protection is concerned.

Another important aspect that was noticed was that of the responsibility for environmental problems, the provided answers placing citizens on the first place in this ranking. By taking responsibility as citizens and not by escalating it to other bodies and institutions, by raising awareness of the importance of the role played by each and every one of us, we could create better conditions for the environmental protection.

Taking an intermediate place in the principle of the 3Rs that aims at reducing the impact of human activities on the environment, as behavior of sustainable consumption, reuse, along with reduction and recycling, can help optimize the use of available resources and, in turn, reduce the carbon footprint. The study has also highlighted the existence of these behaviors in the study population, more than half of the respondents choosing the “You repair the damaged items whenever possible” followed by “You reuse the gift bags and boxes you receive” and “You donate the clothes you no longer use.” Nevertheless, behaviors regarding the benefits of reuse that the population must be informed about have also been identified. A good example is “You donate old but still functional devices to schools or other institutions (computers, laptops)”, where the obtained percentages lead rather to the idea of the absence of this behavior.

The selective waste collection, as part of recycling, was another aspect that this study has tackled, the results indicating the presence of this behavior for the plastic containers, followed by the collection of paper and glass. For these behaviors, the score of 50% was exceeded by cumulating the “always” and “often” answer variants. This is another case where situations that do not indicate the existence of selective collection behaviors have been identified. For “You selectively collect used cooking oil and take it to collection centers” and “You selectively collect printer toner,” the highest values were recorded for the “never” and “rarely” answer variants. Therefore, even for these situations, it would be useful to inform the population about the benefits of this behavior.

The study has also pointed out the fact that, undoubtedly, it is necessary to inform the population about the color codes used on the recycling bins that indicate the type of waste to be collected into them. Although initially three-fourths of the respondents stated that they knew what type of waste should go into which container depending on the bin color, when further assessing their knowledge on this issue, the figures indicated a lower level of information, the range of correct answers being between 45.3% for the brown recycling bins and up to 55.7% for glass.

In order to achieve the objectives and targets regarding the amount of selectively collected waste, in Romania, there was and still is necessary to have a coherent and sustainable legislation, which will lead to, besides the elimination of these environmental pollution factors, to the recycling and reuse of an important part of the waste, as raw or secondary materials (Mihai, 2018; Teodor et al., 2020).

Another solution would be to apply blockchain technology in the waste management field, the aim being to fight climate change for a more sustainable development, but also to comply with the European Union regulations. This technology can lead to a reduction in resource consumption, providing transparency and traceability in the efficient management of the product origin. New digital technologies such as IoT, blockchain technology, AI, etc. combined with circular economy would lead to a greater transparency in waste management and, thus, to the reduction of environmental pollution (Stankovic and Gupta, 2017; Dubey et al., 2019; Geng et al., 2019; Bag et al., 2020; Giudice, 2021; Upadhyay et al., 2021; Yildizbasi, 2021).

## Conclusion

A first solution to remedy the waste problem would be to carry out awareness and education campaigns for university students. By providing theoretical knowledge and by carrying out extracurricular activities, universities can contribute to cultivating environmentally responsible mentalities that lead to the adoption of sustainable habits. The academic world should start to focus more and more on the concept of circular economy by developing new courses and teaching materials in order to provide the students with the skills required by the circular economic model and as a way to achieve sustainable development. In addition, universities play an essential role in shaping the mentalities of professionals, who will occupy key positions in society after completing their studies, being vectors for the multiplication of environmental protection behaviors. As it has already been mentioned, the provision of theoretical knowledge could be done with the consent of the management of higher education institutions by introducing topics for discussion that focus on waste management and the 3Rs in some disciplines’ syllabuses. The extra-curricular part could be achieved mainly by involving student organizations in environmental protection actions. These could target the entire student community by carrying out environmental protection campaigns within the university as well as by a more focused approach on the university campus. Supported by the management of educational institutions, with the help of the hostels’ administrators, such awareness campaigns could lead to the adoption of sustainable behaviors.

Therefore, in waste management, it is necessary to literally involve the entire society represented by local and central public authorities with a decision-making role, by waste generators with a role of reducing quantities and recycling, by professional associations (with a coordinating role at the national level) and research institutions (statistics and forecasts), and by the civil society, actively involved through NGOs or its personnel (the goods consumer and non-governmental organizations).

Although the study has identified gender differences in that women are more aware and more involved in environmental protection activities, regardless of gender, the younger generation should deepen their knowledge in this field and adopt behaviors that could lead to a more sustainable future.

Moreover, by raising awareness and assessing the influences that our behaviors have on the environment, through education, by adopting a sustainable lifestyle and sustainable production and consumption practices, we will be able to reduce the pressure on the planet’s resources. Hopefully, in this way, we will not be coerced into asking the earth for more resources than it can generate, not overusing them in advance and not consuming the natural resources that belong to future generations.

Taking into account the fact that Romania still has a lot of problems to solve in the field of environmental protection, it is possible that this solution provided by the blockchain technology, which is still in full development, will help to solve these issues faster. This technology has the potential to change social behaviors, involving more stakeholders, especially the citizens, boosting the waste management process and leading to the ultimate goal, that of “zero pollution” cities.

Although the study provides some answers regarding the environmental problems we face in Romania, the importance and the identification of the people responsible for solving them, but also regarding the behaviors of reuse and recycling, there are limitations to the undertaken analysis, these being the area of coverage, the fact that only the perspective of the students of the Politehnica University of Timisoara is presented. Further studies need to be carried out on larger geographical areas and on different categories of public, where other variables that can influence the attitudes toward environ-mental protection and the behaviors of reuse and recycling (background of origin, place of origin, the manner in which the legislation in the field is applied, standard of living, economic condition, local culture and beliefs, details about housing conditions, etc.) can be introduced. This objective could be achieved by further conducting qualitative analyses that will definitely lead to a better understanding of the studied behavior.

## Data Availability Statement

The original contributions presented in the study are included in the article/supplementary material, further inquiries can be directed to the corresponding author.

## Ethics Statement

Ethical review and approval was not required for the study on human participants in accordance with the local legislation and institutional requirements. Written informed consent for participation was not required for this study in accordance with the national legislation and the institutional requirements.

## Author Contributions

All authors listed have made a substantial, direct, and intellectual contribution to the work, and approved it for publication.

## Conflict of Interest

The authors declare that the research was conducted in the absence of any commercial or financial relationships that could be construed as a potential conflict of interest.

## Publisher’s Note

All claims expressed in this article are solely those of the authors and do not necessarily represent those of their affiliated organizations, or those of the publisher, the editors and the reviewers. Any product that may be evaluated in this article, or claim that may be made by its manufacturer, is not guaranteed or endorsed by the publisher.
